# Risk factors associated with cisplatin‐induced ototoxicity in Japanese patients with solid tumors

**DOI:** 10.1002/cam4.5565

**Published:** 2022-12-25

**Authors:** Hideaki Okada, Koichi Kitagawa

**Affiliations:** ^1^ Division of Medical Oncology Kobe Minimally Invasive Cancer Center Kobe Japan

**Keywords:** chemotherapy, cisplatin, Japan, ototoxicity, risk factor

## Abstract

**Background:**

Cisplatin, a first‐generation platinum agent, is used for managing various cancers and is associated with dose‐dependent side effects of hearing impairment and tinnitus. However, the safety of high‐dose cisplatin in hearing impairment, has not been fully investigated in Japan.

**Methods:**

We performed pure‐tone threshold audiometry before and every 3–4 weeks after chemotherapy for patients receiving cisplatin‐containing chemotherapy between April 2015 and October 2017 at Kobe Minimally Invasive Cancer Center. Hearing impairment was evaluated prospectively using the National Cancer Institute Common Terminology Criteria for Adverse Events version 4.0.

**Results:**

We enrolled 100 patients and analyzed 96 patients for whom post‐chemotherapy audiometry could be performed. The median patient age was 65 years, and most patients were male (75). The cancer types were as follows: esophageal, 36; head and neck, 35; lung, 23; and gastric, 2. Cisplatin monotherapy and combination therapy were administered to 33 and 63 patients, respectively. A single cisplatin dose was 60–100 mg/m^2^; the median number of doses and total dose were 3 and 240 mg/m^2^, respectively. Additionally, 78 and 18 patients were treated with concurrent chemoradiotherapy and chemotherapy alone, respectively. Twenty‐seven patients had grade 2 or higher hearing impairment. Furthermore, the prevalence was significantly higher in patients receiving a total dose of ≥300 mg/m^2^. Twenty and 32 patients were aware of deafness and tinnitus, respectively.

**Conclusion:**

No patient discontinued treatment owing to hearing impairment. The total cisplatin dose was considered related to post‐treatment hearing impairment frequency in Japanese patients. However, routine audiometric monitoring is recommended during high‐dose cisplatin‐based chemotherapy.

## Graphical abstract

The distribution of the total dose of cisplatin and ototoxicity. The frequency of hearing impairment of CTCEA grade 2/3 was 4.5%/4.5% for the totaldoss of cisplatin for 100‐199, 7.1%/14.3% for 200‐299, and 9.5%/35.7% for 300 or higher. The frequency of grade 2 or higher hearing impairment was more frequent at doses of 300 mg or higher.

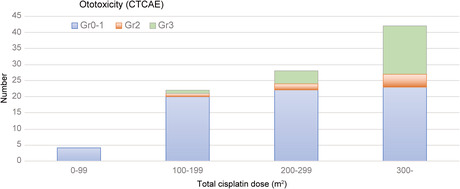

## 1 INTRODUCTION

Cisplatin is a first‐generation platinum‐based effective cytotoxic agent that is currently a standard treatment for a variety of solid tumors, such as non‐small cell lung cancer, head and neck cancer, esophageal cancer, and germ cell tumors. It has a radiation‐sensitizing effect, and when used in combination with radiation therapy, it improves local control and survival outcomes. The serious dose‐limiting adverse events associated with cisplatin include nephrotoxicity, ototoxicity, myelosuppression, and neurotoxicity. In the daily practice of cisplatin use, interventions have been ineffective in preventing ototoxicity in adults. The mechanism of cisplatin‐induced ototoxicity involves the degeneration of the outer hair cells in the organ of Corti and the vascularized epithelium in the lateral wall of the cochlea. The outer hair cells are injured before the inner hair cells, and thus, cisplatin‐induced damage occurs from the base (high frequency) to the apex (low frequency).^1^, ^2^ Long‐term accumulation of cisplatin in the cochlea can explain the delayed progression of cisplatin‐induced hearing loss both preclinically^3^ and clinically.^4^ Peripheral neuropathy due to cisplatin toxicity develops in a dose‐dependent manner. Hearing impairment and tinnitus, which are also dose‐dependent, occur at a high frequency and are the first to occur in cisplatin‐treated patients. The severity of ototoxicity appears to be related to a high cumulative dose, young or old age, pre‐existing hearing impairment, and noise exposure.^5^ The frequency of ototoxicity following cisplatin‐based regimens ranged from 11% to 97% in different studies.^6^, ^7^

Cisplatin is administered at a dose of 60–80 mg/m^2^ for the management of lung cancer,^8^ esophageal cancer,^9^ and gastric cancer^10^; however, a higher dose of 100 mg/m^2^ is recommended for managing head and neck cancer.^11^, ^12^ There have been some reports of cisplatin being highly toxic and difficult to administer in Japan; on the other hand, other studies have reported that it can be administered safely.^13^, ^14^ However, the safety of high‐dose cisplatin administration, especially with regard to hearing impairment, has not been fully investigated. Therefore, we conducted this prospective observational study to identify the onset, frequency, and degree of cisplatin‐induced hearing impairment.

## 2 METHODS

This observational study included patients who received chemotherapy with cisplatin at doses higher than 60 mg/m^2^ at Kobe Minimally Invasive Cancer Center between April 2015 and October 2017. The inclusion criteria were patients with histologically proven malignant tumors who were scheduled to receive cisplatin at a dose higher than 60 mg/m^2^, those older than 20 years of age, and those who could be evaluated regularly using standard pure‐tone audiometry. Exclusion criteria were patients who were unable to undergo regular audiometry tests and were under 20 years of age. All the patients provided written informed consent for participating in the study. The study was approved by the local ethics committee of Kobe Minimally Invasive Cancer Center (approval number: 2015‐study03‐14) and conducted according to the Declaration of Helsinki.

Hearing impairment was evaluated using audiograms, and pure‐tone audiometric thresholds in decibel hearing levels were obtained through air condition at frequencies of 0.5, 1, 2, 3, 4, 6, and 8 kHz. A base hearing evaluation was performed before cisplatin administration and was repeated 3–4 weeks after each cisplatin administration and 1 month after the last administration. Ototoxicity was evaluated using the National Cancer Institute Common Terminology Criteria for Adverse Events (CTCAE), version 4.0, 2010. The primary endpoint was the frequency of hearing impairment of CTCAE grade 2 or higher, and the side with the strongest degree of impairment in each ear was adopted. The secondary endpoint was safety.

Univariate and multivariate analyses were performed to assess risk factors for hearing loss by using Fisher's exact test and logistic regression. Statistical analysis was performed using EZR software, version 1.55 (Saitama Medical Center, Jichi Medical University).^15^ All tests were two‐sided, and *p*‐values of less than 0.05 were considered significant.

## 3 RESULTS

**FIGURE 1:**
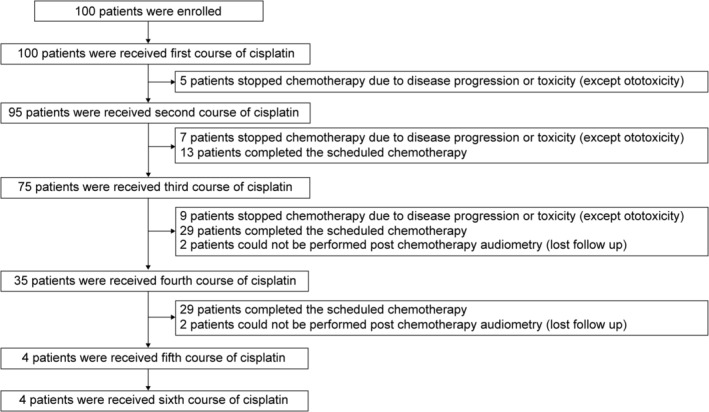
Schematic of patient enrollment and follow‐up

We enrolled 100 patients and analyzed 96 patients for whom hearing tests could be performed after the final administration of cisplatin. The schematic of enrollment and follow‐up is shown in Figure 1. Patient characteristics are shown in Table 1. The median age of the patients was 65 years (range: 41–83 years). Seventy‐five patients were male and 21 were female. The numbers of patients with esophageal, head and neck, lung, and gastric cancers were 36, 35, 23, and 2, respectively. Thirty‐three patients received cisplatin monotherapy, and 63 received cisplatin combination therapy. A single dose of cisplatin ranged from 60 to 100 mg/m^2^, the number of cycles ranged from one to six, and the median cumulative dose of cisplatin was 240 mg/m^2^ (range, 60–540 mg/m^2^). Seventy‐eight patients were treated with radiation therapy, and 18 were treated with chemotherapy alone.

**TABLE 1:** Patient characteristics

Characteristics	(*n* = 96) (%)
Age	
Median	65
Range	41–83
Sex	
Male	75 (78)
Female	21 (22)
Tumor type	
Esophageal cancer	36 (38)
Head and neck cancer	35 (36)
Lung cancer	23 (24)
Gastric cancer	2 (2)
Chemotherapy	
Cisplatin alone	33 (34)
Cisplatin combination	63 (66)
Radiation therapy	
Yes	78 (81)
No	18 (19)
Cisplatin starting dose	
60 mg/m^2^	17 (18)
70 mg/m^2^	23 (24)
75 mg/m^2^	12 (12)
80 mg/m^2^	16 (17)
100 mg/m^2^	28 (29)
Chemotherapy frequency	
1	5 (5)
2	20 (21)
3	38 (39)
4	29 (30)
6	4 (4)
Cisplatin total dose	
Median	240
Range	60–540
<300	54 (56)
≥300	42 (44)

**FIGURE 2:**
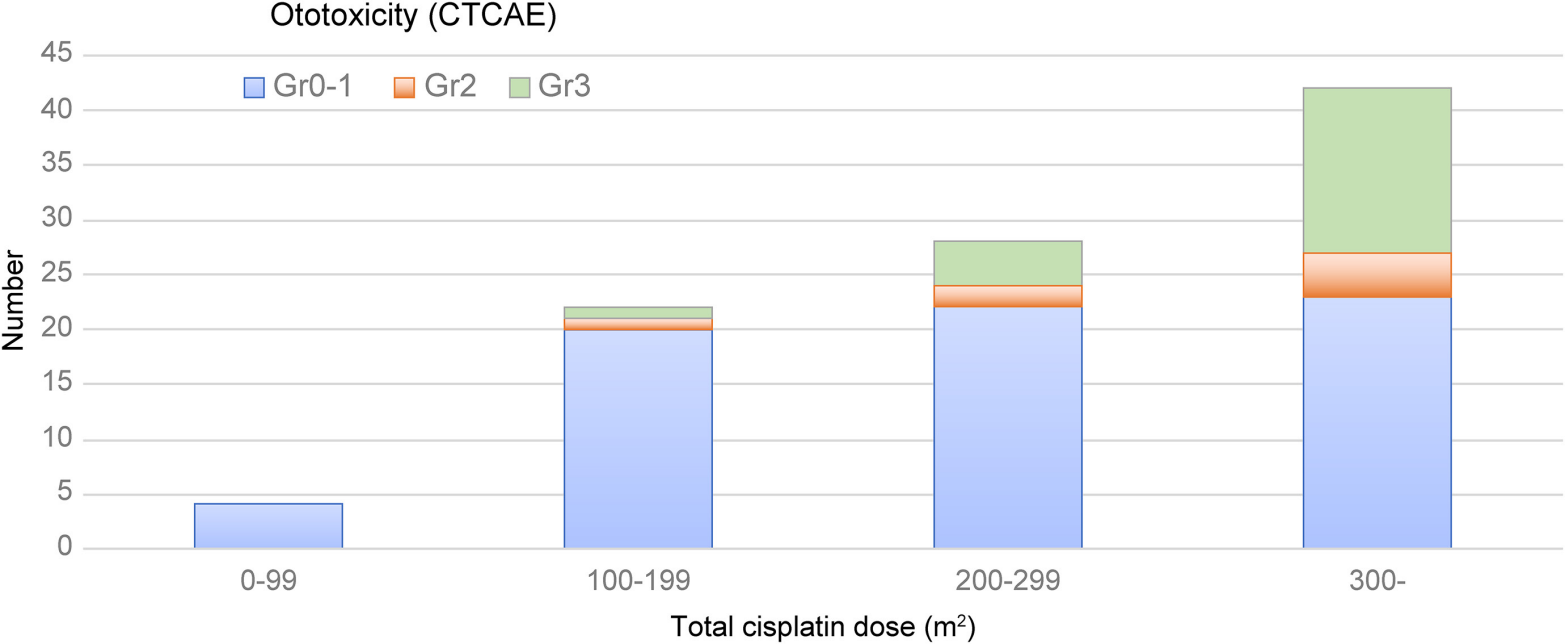
The distribution of the total dose of cisplatin and ototoxicity

The frequency of hearing impairment of CTCAE grade 2 or higher was 28.1% (*n* = 27). Twenty patients (20.8%) were aware of hearing loss, and 32 (33.3%) complained of tinnitus (Table 2). The distribution of the total dose of cisplatin and frequency of grade 2 and grade 3 hearing impairments are shown in Figure 2. In cases wherein a single dose of 100 mg/m^2^ was administered, hearing impairment of grade 2 or higher was observed in more than 50% of patients after two cycles of cisplatin administration. On the other hand, in cases in which the cisplatin dose administered was 80 mg/m^2^ or less, hearing impairment was noted in 20% of patients after four cycles of administration (Table 3). Multivariate analysis was performed to determine the risk factors for hearing impairment of grade 2 or higher. A total dose of ≥300 mg/m^2^ (odds ratio, 3.130; *p* = 0.042) was a significantly associated factor (Table 4).

**TABLE 2:** Results of audiometry

	(*n* = 96) (%)
Hearing impairment (CTCAE v4.0)	
Grade 0	42
Grade 1	27
Grade 2	7
Grade 3	20
≥Grade 2	27 (28.1)
Awareness of hearing impairment	
Yes	20 (20.8)
No	76
Awareness of tinnitus	
Yes	32 (33.3)
No	64

**TABLE 3:** Time of emergence of grade 2 or higher hearing impairment

CDDP dose (mg/m^2^)	After 1 cycle	After 2 cycles	After 3 cycles	After 4 cycles
60	1/17	1/15	1/12	2/10
(*N* = 17)	(5.8%)	(6.7%)	(8.3%)	(20%)
70–80	1/51	5/50	5/33	5/23
(*N* = 51)	(2.0%)	(10.0%)	(15.2%)	(21.7%)
100	3/28	14/27	14/26	1/2
(*N* = 28)	(10.7%)	(51.9%)	(53.8%)	(50.0%)

**TABLE 4:** Univariate and multivariate analyses of grade 2 or higher hearing impairment

		Univariate analysis		Multivariate analysis	
		Odds ratio (95% CI)	*p*‐value	Odds ratio (95% CI)	*p*‐value
Sex	Male	0.555 (0.178–1.794)	0.278	0.440 (0.132–1.47)	0.182
	Female				
Age	<70 years	0.858 (0.264–2.550)	1		
	≥70 years				
Hypertension	Yes	0.749 (0.232–2.204)	0.629		
	No				
Use of furosemide	Yes	0.903 (0.310–2.801)	1		
	No				
Albumin	≥3.5	1.511 (0.354–9.173)	0.750		
	<3.5				
Chemotherapy frequency	≥4	0.938 (0.319–2.615)	1		
	<4				
CDDP starting dose	≥100	5.270 (1.831–15.86)	0.001	2.690 (0.845–8.54)	0.093
	<100				
Radiation therapy	Yes	3.731 (0.779–35.96)	0.087	2.950 (0.534–16.30)	0.215
	No				
Total CDDP dose	≥300	4.666 (1.653–14.33)	0.001	3.130 (1.040–9.40)	0.042
	<300				

## 4 DISCUSSION

To the best of our knowledge, this is the first study to evaluate the risk factors of cisplatin‐induced hearing impairment in adult Japanese patients with cancer. Most reports of cisplatin‐induced hearing impairment in children are from Europe and the United States. The strength of this study is that it prospectively examined the frequency and timing of hearing impairment and risk factors using the CTCAE, which is widely used to evaluate the toxicity of chemotherapy in Japanese patients.

The frequency of hearing impairment due to cisplatin is 11–97%, which varies considerably from report to report. This may be owing to the fact that there are various ways to assess hearing impairment and the lack of international consensus on the evaluation method. The Brock classification^16^ and Muenster classification^17^ are used for hearing impairment evaluations in children. However, this study was conducted using the NCI CTCAE, which is often used to assess the toxicity of chemotherapy in adults. The frequency of grade 2 or higher hearing impairment was 28.1%, which is consistent with that in previous reports.^18^, ^19^

In a study comparing doses and schedules for lung cancer, hearing impairment after cisplatin administration was predominantly observed at high administration frequencies and at doses of ≥60 mg/m^2^.^20^ In children with hepatoblastoma, the incidence of hearing impairment was reduced by the administration of sodium thiosulfate 6 h after cisplatin chemotherapy^21^; however, the efficacy of this approach in adults is unclear. Hearing impairment is basically irreversible, and it is necessary to perform hearing tests regularly and consider whether or not to continue cisplatin use depending on the symptoms. Ototoxicity monitoring guidelines recommend regular hearing tests; however, adherence to this recommendation is poor.^22^, ^23^

Several studies have identified the total dose of cisplatin as an important predictor of cisplatin‐induced hearing impairment. The cutoff cumulative dose of cisplatin has been reported as 200–400 mg/m^2^.^23^ In line with the reported dose, in our study, the frequency of hearing impairment of a grade higher than 2 was significantly associated with the administration of a dose of 300 mg/m^2^ or higher.

In a report examining a single dose of cisplatin, the frequency of hearing impairment was significantly higher in patients receiving a dose of 100 mg/m^2^ every 3 weeks than in those receiving a weekly dose of 30 mg/m^2^.^24^ In a study of postoperative chemoradiation for head and neck cancer in Japan, the frequency of hearing impairment was higher in the every 3 weeks group than in the weekly group.^25^ In the TAX323 study wherein cisplatin was administered every 3 weeks, the frequency of grade 3 or higher hearing impairment was 2.8% in the cisplatin plus fluorouracil group (cisplatin 100 mg/m^2^) and 0% in the cisplatin and fluorouracil plus docetaxel group (cisplatin 75 mg/m^2^).^26^ Higher initial doses of cisplatin appeared to be associated with an increase in hearing impairment.^27^, ^28^, ^29^ In our study, the frequency of hearing impairment tended to be higher in cases in which a single dose of 100 mg/m^2^ was administered than in those in which a single dose of 80 mg/m^2^ or less was used, and impairment occurred early in the former group of cases.

Our study has some limitations. First, the sample size was small, and there is a risk of bias due to the observational nature of the study. Second, although various cancers were targeted, we only included patients diagnosed with head and neck, gastrointestinal, and lung cancers. Therefore, little data were available for young patients. Third, because we targeted a single dose of cisplatin, we could not examine cases with divided doses. Fourth, late audiometric data were not available to evaluate the late ototoxic effects of cisplatin.

## 5 CONCLUSIONS

In conclusion, no cases of discontinued chemotherapy owing to hearing impairment were noted; therefore, high‐dose cisplatin could be administered to Japanese patients. To the best of our knowledge, this study is the first to confirm this finding in patients with esophageal and gastric cancer; previous studies have reported similar findings only in patients with head, neck, and lung cancer.

In cases of head and neck cancer in which 100 mg/m^2^ of cisplatin is administered or in patients receiving total doses higher than 300 mg/m^2^, the risk of hearing impairment is considered to be high. Therefore, a pretreatment audiogram and routine audiometric monitoring should be performed during high‐dose cisplatin‐based chemotherapy.

## AUTHOR CONTRIBUTIONS

**Hideaki Okada:** Conceptualization (equal); data curation (equal); formal analysis (equal); investigation (equal); writing – original draft (equal); writing – review and editing (equal). **Koichi Kitagawa:** Data curation (equal); investigation (equal); writing – review and editing (equal).

## CONFLICT OF INTEREST

The authors declare no conflicts of interest.

## ETHICAL APPROVAL STATEMENT

The study was approved by the local ethics committee of Kobe Minimally Invasive Cancer Center (approval number: 2015‐study03‐14) and conducted according to the Declaration of Helsinki.

## PATIENT CONSENT STATEMENT

All patients provided written informed consent for participation in the study.

## Data Availability

The data that support the findings of this study are available from the corresponding author upon reasonable request.

## References

[1] Brock PR , Knight KR , Freyer DR , et al. Platinum‐induced ototoxicity in children: a consensus review on mechanisms, predisposition, and protection, including a new International Society of Pediatric Oncology Boston ototoxicity scale. J Clin Oncol. 2012;30(19):2408‐2417. doi:10.1200/JCO.2011.39.1110 22547603 PMC3675696

[2] Rybak LP , Whitworth CA , Mukherjea D , Ramkumar V . Mechanisms of cisplatin‐induced ototoxicity and prevention. Hear Res. 2007;226(1–2):157‐167. doi:10.1016/j.heares.2006.09.015 17113254

[3] Breglio AM , Rusheen AE , Shide ED , et al. Cisplatin is retained in the cochlea indefinitely following chemotherapy. Nat Commun. 2017;8(1):1654. doi:10.1038/s41467-017-01837-1 29162831 PMC5698400

[4] Kolinsky DC , Hayashi SS , Karzon R , Mao J , Hayashi RJ . Late onset hearing loss: a significant complication of cancer survivors treated with cisplatin containing chemotherapy regimens. J Pediatr Hematol Oncol. 2010;32(2):119‐123. doi:10.1097/MPH.0b013e3181cb8593 20098336

[5] Chirtes F , Albu S . Prevention and restoration of hearing loss associated with the use of cisplatin. Biomed Res Int. 2014;2014:925485. doi:10.1155/2014/925485 25140325 PMC4129932

[6] Marshak T , Steiner M , Kaminer M , Levy L , Shupak A . Prevention of cisplatin‐induced hearing loss by intratympanic dexamethasone: a randomized controlled study. Otolaryngol Head Neck Surg. 2014;150(6):983‐990. doi:10.1177/0194599814524894 24618499

[7] Fausti SA , Henry JA , Schaffer HI , Olson DJ , Frey RH , Bagby GC Jr . High‐frequency monitoring for early detection of cisplatin ototoxicity. Arch Otolaryngol Head Neck Surg. 1993;119(6):661‐666. doi:10.1001/archotol.1993.01880180081015 8499098

[8] Ohe Y , Ohashi Y , Kubota K , et al. Randomized phase III study of cisplatin plus irinotecan versus carboplatin plus paclitaxel, cisplatin plus gemcitabine, and cisplatin plus vinorelbine for advanced non‐small‐cell lung cancer: four‐arm cooperative study in Japan. Ann Oncol. 2007;18(2):317‐323. doi:10.1093/annonc/mdl377 17079694

[9] Minsky BD , Pajak TF , Ginsberg RJ , et al. INT 0123 (radiation therapy oncology group 94‐05) phase III trial of combined‐modality therapy for esophageal cancer: high‐dose versus standard‐dose radiation therapy. J Clin Oncol. 2002;20(5):1167‐1174. doi:10.1200/JCO.2002.20.5.1167 11870157

[10] Koizumi W , Narahara H , Hara T , et al. S‐1 plus cisplatin versus S‐1 alone for first‐line treatment of advanced gastric cancer (SPIRITS trial): a phase III trial. Lancet Oncol. 2008;9(3):215‐221. doi:10.1016/S1470-2045(08)70035-4 18282805

[11] Adelstein DJ , Li Y , Adams GL , et al. An intergroup phase III comparison of standard radiation therapy and two schedules of concurrent chemoradiotherapy in patients with unresectable squamous cell head and neck cancer. J Clin Oncol. 2003;21(1):92‐98. doi:10.1200/JCO.2003.01.008 12506176

[12] Al‐Sarraf M , LeBlanc M , Giri PG , et al. Chemoradiotherapy versus radiotherapy in patients with advanced nasopharyngeal cancer: phase III randomized intergroup study 0099. J Clin Oncol. 1998;16(4):1310‐1317. doi:10.1200/JCO.1998.16.4.1310 9552031

[13] Isobe K , Kawakami H , Uno T , et al. Concurrent chemoradiotherapy for locoregionally advanced nasopharyngeal carcinoma: is intergroup study 0099 feasible in Japanese patients? Jpn J Clin Oncol. 2003;33(10):497‐500. doi:10.1093/jjco/hyg094 14623916

[14] Kiyota N , Tahara M , Okano S , et al. Phase II feasibility trial of adjuvant chemoradiotherapy with 3‐weekly cisplatin for Japanese patients with post‐operative high‐risk squamous cell carcinoma of the head and neck. Jpn J Clin Oncol. 2012;42(10):927‐933. doi:10.1093/jjco/hys128 22923484

[15] Kanda Y . Investigation of the freely available easy‐to‐use software 'EZR' for medical statistics. Bone Marrow Transplant. 2013;48(3):452‐458. doi:10.1038/bmt.2012.244 23208313 PMC3590441

[16] Brock PR , Bellman SC , Yeomans EC , Pinkerton CR , Pritchard J . Cisplatin ototoxicity in children: a practical grading system. Med Pediatr Oncol. 1991;19(4):295‐300. doi:10.1002/mpo.2950190415 2056973

[17] Schmidt CM , Bartholomäus E , Deuster D , Heinecke A , Dinnesen AG . Die "Münsteraner Klassifikation": Eine neue Einteilung der Hochtonschwerhörigkeit nach Cisplatingabe [the "muenster classification" of high frequency hearing loss following cisplatin chemotherapy]. HNO. 2007;55(4):299‐306. doi:10.1007/s00106-005-1368-1 16437215

[18] Esfahani Monfared Z , Khosravi A , Safavi Naini A , Radmand G , Khodadad K . Analysis of cisplatin‐induced ototoxicity risk factors in Iranian patients with solid tumors: a cohort, prospective and single institute study. Asian Pac J Cancer Prev. 2017;18(3):753‐758. doi:10.22034/APJCP.2017.18.3.753 28441710 PMC5464495

[19] Laurell G , Jungnelius U . High‐dose cisplatin treatment: hearing loss and plasma concentrations. Laryngoscope. 1990;100(7):724‐734. doi:10.1288/00005537-199007000-00008 2362532

[20] Rademaker‐Lakhai JM , Crul M , Zuur L , et al. Relationship between cisplatin administration and the development of ototoxicity. J Clin Oncol. 2006;24(6):918‐924. doi:10.1200/JCO.2006.10.077 16484702

[21] Brock PR , Maibach R , Childs M , et al. Sodium thiosulfate for protection from cisplatin‐induced hearing loss. N Engl J Med. 2018;378(25):2376‐2385. doi:10.1056/NEJMoa1801109 29924955 PMC6117111

[22] Whitehorn H , Sibanda M , Lacerda M , et al. High prevalence of cisplatin‐induced ototoxicity in Cape Town, South Africa. S Afr Med J. 2014;104(4):288‐291. doi:10.7196/samj.7389 25118554

[23] Santucci NM , Garber B , Ivory R , Kuhn MA , Stephen M , Aizenberg D . Insight into the current practice of ototoxicity monitoring during cisplatin therapy. J Otolaryngol Head Neck Surg. 2021;50(1):19. doi:10.1186/s40463-021-00506-0 33766142 PMC7995701

[24] Noronha V , Joshi A , Patil VM , et al. Once‐a‐week versus once‐every‐3‐weeks cisplatin chemoradiation for locally advanced head and neck cancer: a phase III randomized noninferiority trial. J Clin Oncol. 2018;36(11):1064‐1072. doi:10.1200/JCO.2017.74.9457 29220295

[25] Kiyota N , Tahara M , Mizusawa J , et al. Weekly cisplatin plus radiation for postoperative head and neck cancer (JCOG1008): a multicenter, noninferiority, phase II/III randomized controlled trial. J Clin Oncol. 2022;40(18):1980‐1990. doi:10.1200/JCO.21.01293 35230884 PMC9197353

[26] Vermorken JB , Remenar E , van Herpen C , et al. Cisplatin, fluorouracil, and docetaxel in unresectable head and neck cancer. N Engl J Med. 2007;357(17):1695‐1704. doi:10.1056/NEJMoa071028 17960012

[27] Schmitt NC , Page BR . Chemoradiation‐induced hearing loss remains a major concern for head and neck cancer patients. Int J Audiol. 2018;57(sup4):S49‐S54. doi:10.1080/14992027.2017.1353710 28728452 PMC6119124

[28] Theunissen EA , Bosma SC , Zuur CL , et al. Sensorineural hearing loss in patients with head and neck cancer after chemoradiotherapy and radiotherapy: a systematic review of the literature. Head Neck. 2015;37:281‐292. doi:10.1002/hed.23551 24478269

[29] Teft WA , Winquist E , Nichols AC , et al. Predictors of cisplatin‐induced ototoxicity and survival in chemoradiation treated head and neck cancer patients. Oral Oncol. 2019;89:72‐78. doi:10.1016/j.oraloncology.2018.12.010 30732962

